# Role of peritoneal washing cytology in ovarian malignancies: correlation with histopathological parameters

**DOI:** 10.1186/s12957-015-0732-1

**Published:** 2015-11-10

**Authors:** Samreen Naz, Atif Ali Hashmi, Rabia Ali, Naveen Faridi, Syed Danish Hussian, Muhammad Muzzammil Edhi, Mehmood Khan

**Affiliations:** Department of Histopathology, Liaquat National Hospital and Medical College, Karachi, Pakistan; Liaquat National Hospital and Medical College, Karachi, Pakistan; Dhaka Medical College, Dhaka, Bangladesh

**Keywords:** Peritoneal washing, Ovarian cancer, Omental metastasis

## Abstract

**Background:**

Peritoneal dissemination of ovarian tumors is a major prognostic parameter in ovarian malignancies. Analysis of peritoneal washing cytology serves as a useful predictor of ovarian surface involvement and peritoneal metastasis even in the absence of clinical omental spread. The aim of the current study is to correlate peritoneal cytology with various histologic features of ovarian cancers in our setup.

**Methods:**

A total of 60 cases of ovarian tumors were included in the study that underwent total abdominal hysterectomy with bilateral salpingo-oophorectomy and omental and lymph node sampling during 2009 till 2014 at the Liaquat National Hospital, Karachi. Any free abdominal fluid was aspirated at the time of surgery. In the absence of free fluid, peritoneal washing was done with 50–100 ml of normal saline. Four cytospin preparations were done along with a cell block preparation. Correlation of peritoneal cytology with various histologic parameters was performed.

**Results:**

Out of the 60 cases of ovarian tumors involved in the study, 56 were surface epithelial tumors, 2 germ cell tumors, and 2 metastatic carcinomas. The mean tumor size was 9.6 cm. Capsular invasion was seen in 61 % of the cases, and omental metastasis in 51 % of the cases. Serous carcinoma was found to have a significantly higher frequency of positive peritoneal cytology (76.9 %) compared to endometrioid and mucinous carcinomas (44 and 25 %, respectively). A significant positive correlation was seen between positive peritoneal cytology and capsular invasion and omental metastasis with a *p* value of <0.001.

**Conclusions:**

Positive peritoneal washing cytology has been implemented in ovarian cancer guidelines because of its prognostic significance in ovarian tumors. In addition to being an indicator of peritoneal metastasis, positive cytology also correlates with capsular invasion and histologic type in ovarian tumors. Therefore, it should always be used as an adjunctive tool in the surgical management of ovarian tumors.

## Background

Ovarian cancer is a significant cause of morbidity and mortality in women in this part of the world due to lack of any effective screening protocol. According to Karachi cancer registry, ovarian cancer represents the third most common malignancy in Karachi with serous adenocarcinoma being the most common (33.3 %) [1]. Ovarian cancers are usually detected late in the disease course when it is significantly enlarged in size to cause abdominal distention and distress. However, despite of being of large sizes, ovarian tumors may still be confined to the ovaries. On the other hand, omental and peritoneal spread of ovarian cancers has a huge impact on prognosis and upstages prognostic morbidity. Analysis of peritoneal washing cytology (PWC) serves as a useful predictor of ovarian surface involvement and peritoneal metastasis in ovarian cancers [2, 3]. It can also detect subclinical peritoneal spread of the disease. While the procedure is routinely done in our institution as part of the protocol for all surgeries of suspected ovarian malignancies, its usefulness has not been objectively evaluated. The aim of the current study is to correlate peritoneal cytology with various histologic features of ovarian malignancies.

## Methods

**Fig. 1:**
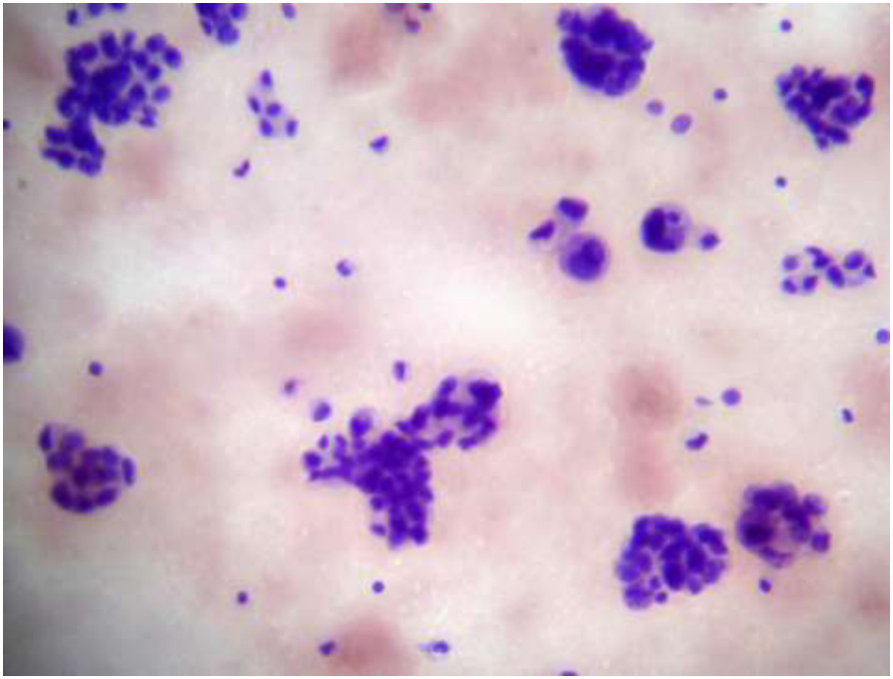
Positive peritoneal cytology in ovarian serous carcinoma

**Fig. 2:**
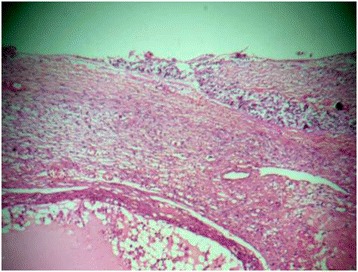
Capsular invasion in ovarian carcinoma

The study was approved by the Liaquat National Hospital and Medical College ethical review committee, and written informed consent was obtained from all subjects. A total of 60 cases of borderline and malignant ovarian tumors were included in the study that underwent total abdominal hysterectomy and bilateral salpingo-oophorectomy (TAH with BSO) along with omental and lymph node samplings at the Liaquat National Hospital, Karachi from January 2009 till December 2014. After opening the abdominal cavity, any free fluid was aspirated. Various areas of peritoneal cavity including paracolic gutters were lavaged with 50–100 ml of normal saline and sent for cytologic examination. Four cytospin preparations were stained with hematoxylin and eosin and Papanicolaou stains after fixation along with a cell block preparation. TAH with BSO was performed afterwards with omental and lymph node samplings. All cases undergoing ovarian surgery with peritoneal cytologic examination, omental and lymph node samplings were consecutively enrolled in the study. Pathologists reporting peritoneal cytology were blinded with the surgical pathology findings. Results of peritoneal cytology were correlated with various histologic features of ovarian tumors including histologic type, grade, tumor size, capsular invasion, and omental metastasis (Figs. 1 and 2), using chi-square test (univariate analysis). A *p* value of <0.05 was considered as statistically significant.

## Results

The mean age at diagnosis was 49.9 years (±14.8). Out of the 60 cases of ovarian tumors involved in the study, 56 were surface epithelial tumors, 2 germ cell tumors, and 2 metastatic carcinomas. The mean tumor size was 11.2 cm. Capsular invasion was seen in 61 % of tumors and omental metastasis in 51 % of tumors. Table 1 shows the histopathologic features of ovarian tumors involved in the study. Serous tumors were the most frequent histologic subtype followed by mucinous and endometrioid tumors. Serous carcinoma had a significantly higher frequency of positive peritoneal cytology (76.9 %) compared to endometrioid and mucinous carcinomas (44 and 25 %, respectively) as shown in Table 2. A significant positive correlation was seen between positive peritoneal cytology with omental metastasis and capsular invasion with a *p* value of <0.001 (Tables 3 and 4). Higher grade tumors were more likely to have positive peritoneal cytology than low-grade tumors; however, the *p* value was not found to be statistically significant (Table 5). Tumors of smaller sizes tend to have a significantly higher incidence of positive peritoneal cytology (Table 6).

**Table 1:** Histopathologic features of ovarian tumors and its correlation with peritoneal cytology

	Frequency	Omental metastasis	Capsular invasion	Tumor size	
		Frequency(%)	Frequency(%)	Frequency(%)	Mean
Serous tumors	27				11.8
Malignant	26	20(76.9)	19(73.1)	22(84.6)	8.6
Borderline	1	0(0)	0(0)	0(0)	15
Mucinous tumors	12				22.6
Malignant	8	2(25)	2(25)	2(25)	24.8
Borderline	4	0(0)	0(0)	1(25)	20.5
Clear cell carcinoma	1	1(100)	1(100)	1(100)	7
Transitional cell carcinoma	2	2(100)	2(100)	1(50)	8
Malignant mixed mullerian tumor	3	2(66.7)	1(33.3)	3(100)	9.3
Endometrioid carcinoma	9	4(44.4)	2(22.2)	3(33.3)	6.5
Metastatic carcinoma	2	1(50)	1(50)	1(50)	3.5
Dysgerminoma	2	1(50)	1(50)	1(50)	11
Poorly differentiated adenocarcinoma	2	2(100)	2(100)	2(100)	7
Total	60	35(58.3)	31(51.6)	37(61.6)	9.65

**Table 2:** Correlation of peritoneal cytology with histological subtype of tumor

	Peritoneal.cytology				*p* value
	Positive		Negative		
	Frequency	%	Frequency	%	
Serous carcinoma	20	76.92	6	23.08	0.042
Endometrioid carcinoma	4	44.44	5	55.56	
Mucinous carcinoma	2	25.00	6	75.00	
Clear cell carcinoma	1	100.00	0	0.00	
Transitional cell carcinoma	2	100.00	0	0.00	
Malignant mixed mullerian tumor	2	66.67	1	33.33	
Borderline serous tumor	0	0.00	1	100.00	
Borderline mucinous tumor	0	0.00	4	100.00	
Metastatic carcinoma	1	50.00	1	50.00	
Dysgerminoma	1	50.00	1	50.00	
Poorly differentiated adenocarcinoma	2	100.00	0	0.00	
Total	35	58.33	25	41.67

**Table 3:** Correlation of peritoneal cytology with capsular invasion

Peritoneal cytology	Frequency(%)	capsular.invasion		*p* value
		Present	Absent	
Positive	35(58.3)	30(85.7)	5(14.3)	<0.001
Negative	25(41.6)	7(28.0)	18(72.0)	
Total	60	37(61.7)	23(38.3)

**Table 4:** Correlation of Peritoneal cytology with Omental Metastasis

Peritoneal cytology	Frequency(%)	omental.metastasis		*p* value
		Present	Absent	
Positive	35(58.3)	29(82.9)	6(17.1)	<0.001
Negative	25(41.6)	2(8.0)	23(92.0)	
Total	60	31(51.7)	29(48.3)

**Table 5:** Correlation of peritoneal cytology with tumor grade

Peritoneal cytology	Tumor grade			Total	*p* value
	Low grade	High grade	Intermediate		
Positive	1(20 %)	30(66.6 %)	4(40 %)	35(58.3 %)	
Negative	4(80 %)	15(33.3 %)	6(60 %)	25(41.6 %)	0.058
Total	5(8.3 %)	45(75 %)	10(16.6 %)	60

**Table 6:** Correlation of peritoneal cytology with tumor size

Peritoneal cytology	Tumor size			Total	*p* value
	<10 cm	10–20 cm	>20 cm		
Positive	28(84.8 %)	6(31.5 %)	1(12.5 %)	35(58.3 %)	
Negative	5(15.1 %)	13(68.4 %)	7(87.5 %)	25(41.6 %)	<0.001
Total	33(55 %)	19(31.6 %)	8(13.3 %)	60	

## Discussion

Peritoneal washing cytology is a useful indicator of ovarian surface involvement and peritoneal dissemination by ovarian tumors. It may identify subclinical peritoneal spread and thus provide valuable staging and prognostic information [4]. For the same reason, PWC was implemented in ovarian cancer guidelines and is routinely performed in ovarian cancer surgeries.

In our study, we report our experience of PWC with emphasis over its correlation with histological parameters, i.e., tumor type, tumor grade, tumor size, capsular invasion, and omental metastasis.

Cytology detection rates of abdominal spread in ovarian cancers vary in different studies. Fadare et al. reported 25 % and Rubin et al. reported 30 % detection rate, while Colgan et al. revealed 50 % detection rate [5–7]. As much as 90 % positive cytology detection rate has also been reported in a study [8]. This can be due to the inclusion of ascitic fluid along with peritoneal washings, as ascitic fluid has a much higher rate of detecting malignant cells.

In our observation, serous carcinomas (*n* = 26.79 %) were most likely and mucinous carcinoma (*n* = 8, 25 %) were least likely to involve abdominal cavity as represented by positive peritoneal cytology. Forty-four percent of endometrioid carcinoma (*n* = 9), 100 % of clear cell carcinoma (*n* = 1), 100 % of transitional cell carcinoma (*n* = 1), 66 % of malignant mullerian tumors (*n* = 3), and 50 % of germ cell tumors (*n* = 2) showed positive cytology. Fadare et al. reported 71.4 % of serous carcinoma (*n* = 57), 55 % of endometrioid carcinoma (*n* = 30), 20 % of clear cell carcinoma (*n* = 19), and 50 % of mucinous carcinoma (*n* = 13) showing positive peritoneal cytology [5]. Similar study by Rubin et al. analyzed 96 cases of ovarian tumors of which 29 cases showed positive PWC [6]. The histological subtypes included the following: serous, 21 of 66 (32 %); endometrioid, 3 of 11 (27 %); mucinous 0 of 1 (0 %); and clear cell carcinoma, 2 of 3 (66 %).

In patients with ovarian serous tumor of low malignant potential and borderline tumors, PWC is a relatively sensitive indicator for presence of peritoneal implants whether invasive or non-invasive [2]. Positive cytology upstages borderline tumors. Mulvany, in his study, concluded that the clinical value of PWC lies in upstaging 63 % of borderline ovarian tumors [3]. Cheng et al. demonstrated a 44 % positive cytology detection rate of borderline mucinous tumors [9]. However, in our study, there were only five borderline tumor cases (one serous, four mucinous). All showed negative cytology as well as no omental metastasis.

Omental metastasis/peritoneal histology are used as a standard to determine the sensitivity and specificity of PWC. In our study, 35 cases showed positive PWC and 31 showed omental metastasis. There were six cases (6/35; 17 %) that were cytology positive and histology negative. These included two serous carcinomas, two endometrioid carcinoma, one mucinous carcinoma, and one MMT. Out of 25 cytology negative cases, only two showed omental metastasis (2/25; 8 %), one serous, and one mucinous. Sneige et al., in their study, observed that 11/70 cases (16 %) were cytology positive and biopsy negative [2]. In a study by Zuna and Behrens, 112 ovarian carcinoma cases were analyzed, and only three stage I cases had positive cytology and negative peritoneal histology (two carcinomas and one borderline tumor) [10]. Of these, only patients with borderline tumors were alive at the last follow-up. Other two died of the disease. In one study, late recurrences were reported in patients with low-grade serous tumors having negative staging biopsy, and they concluded that this may be due to sampling error at the site of implant [11]. Hence, stage I disease without omental metastasis but positive cytology may indicate increased risk of tumor recurrence, and this emphasizes the importance of PWC. In our study, the correlation of PWC with omental metastasis was statistically significant.

According to the recent Federation of Gynecology and Obstetrics (FIGO) guidelines, capsular rupture upstages a stage I tumor to stage IC2 and positive cytology to stage IC3 [12]. Hence, determination of capsular integrity is important as this changes cancer stage. In our study, we tried to correlate capsular invasion with PWC. Out of 60 cases, 37 showed capsular invasion but not all showed positive cytology. Of cytology-positive cases, only 5 had capsule intact and out of 25 cytology negative cases, and 7 had capsular rupture. Of 26 serous carcinomas, 22 (84 %) showed capsular invasion while 20 (76.9 %) showed positive cytology. One borderline mucinous tumor had capsular invasion but a negative cytology. All three MMT showed ruptured capsule (invasion) with two showing positive PWC. Only one case (endometrioid carcinoma) showed positive cytology with intact capsule. With or without capsular invasion, the presence of PWC in stage I disease alone is sufficient to upgrade the tumor. We observed a strong correlation between capsular invasion and PWC (*p* = <0.001).

In our study, the mean tumor size was different for different tumor types. It was 11.58 cm for serous tumors, 6.5 cm for endometrioid tumors, and 22 cm for mucinous neoplasms. The largest tumor size was 30 cm of mucinous carcinoma. Positive peritoneal cytology was correlated to tumor size, and we observed that most of the positive cytology cases had tumor size of less than 10 cm (28/35 cases, 84 %) and only one tumor of more than 20 cm showed positive cytology. Interestingly, most (7/8) large tumors (>20 cm) had negative cytology. Of these, only one showed omental metastasis, i.e., histology positive. The rest had intact capsules and no omental metastasis. All large tumors were mucinous. The only large tumor with positive cytology was 30 cm in size with capsular invasion and omental metastasis. We observed that mucinous tumors may acquire large sizes but mostly remain organ confined with negative cytology. Some of the intermediate sized serous tumors also showed negative cytology. Hence, smaller tumors have a significantly more incidence of positive PWC.

Tumor grade also had an impact on abdominal spread of ovarian tumors. We found out that 30 out of the 35 cytology-positive cases had high histological grade, four had intermediate grade, and only one had low grade. Fifteen (33 %) of high-grade tumors showed negative histology. Ozkara reported that PW were significantly more likely to yield malignant cells in higher grade than lower grade tumors [13].

## Conclusions

In conclusion, positive peritoneal washing cytology is a useful prognostic factor in ovarian tumors. In addition to being an indicator of peritoneal metastasis, positive cytology also correlates with capsular invasion, tumor type, and tumor grade in ovarian tumors.

## Consent

Written informed consent was obtained from the patients for publication of this article and accompanying images. A copy of the written consent is available for review by the Editor-in-Chief of this journal. The approval was obtained from the Liaquat National Hospital and Medical College ethical review committee.

## Abbreviations

FIGO: Federation of Gynecology and Obstetrics
PWC: peritoneal washing cytology
